# Association between polymorphisms in MicroRNA target sites of *RAD51D* genes and risk of hepatocellular carcinoma

**DOI:** 10.1002/cam4.2068

**Published:** 2019-03-18

**Authors:** Yan‐Ji Jiang, Jian‐Hong Zhong, Zi‐Han Zhou, Mo‐Qin Qiu, Xian‐Guo Zhou, Ying‐Chun Liu, Rong‐Rui Huo, Xiu‐Mei Liang, Zhu Chen, Qiu‐Ling Lin, Xiang‐Yuan Yu, Hong‐Ping Yu

**Affiliations:** ^1^ Affiliated Tumor Hospital of Guangxi Medical University Guangxi China; ^2^ School of Public Health Guangxi Medical University Guangxi China; ^3^ Affiliated Hospital of Guilin Medical University Guangxi China; ^4^ School of Public Health Guilin Medical University Guangxi China

**Keywords:** hepatocellular carcinoma, *RAD51D*, single–nucleotide polymorphism, susceptibility

## Abstract

*RAD51D (RAD51L3)* is a member of the *RAD51* gene family which plays important roles in maintaining genomic stability and preventing DNA damage. This study is aimed to investigate the associations between *RAD51D *polymorphisms and the hereditary susceptibility of hepatocellular carcinoma (HCC). In this study we conducted a hospital–based case‐control study including 805 cases (HCC patients) and 846 controls (nontumor patients) in Guangxi, China. A total of two Single–nucleotide polymorphisms (SNPs) rs12947947 and rs28363292 of *RAD51D *were selected and genotyped. Although we did not find two SNPs individually that had any significant main effect on risk of HCC, We found that the combined genotypes with 1‐2 risk genotypes were associated with significantly increased overall risk of HCC (OR = 1.462, 95% CI = 1.050‐2.036). According to the results of further stratification analysis, GT/GG genotype of rs28363292 increased HCC risk in zhuang people (OR = 3.913, 95% CI = 1.873‐8.175) and nonhepatitis B virus (HBV) infection population (OR = 1.774, 95% CI = 1.060‐2.969), the combined 1‐2 risk genotypes increased the risk of HCC in zhuang people (OR = 2.817, 95% CI = 1.532‐5.182) and non‐HBV infected population (OR = 1.567, 95% CI = 1.042‐2.358). Our results suggest that rs12947947 and rs28363292 polymorphisms may jointly contribute to the risk of HCC. Further large studies and functional studies are required to validate our findings.

## 1 INTRODUCTION

Liver cancer is the fifth most common cancer in the world and the second leading cause of death.^1^, ^2^ Hepatocellular carcinoma (HCC) is the most common primary malignancy of the liver, and it accounts for between 85% and 90% of these malignancies.^3^, ^4^ Each year there are approximately 630 000 new cases of HCC in the world and more than half of the new cases occur in China alone.^5^ HCC has been regarded as a complicated disease caused by factors including hepatitis B virus (HBV) or hepatitis C virus (HCV), tobacco use, and alcohol consumption.^6^, ^7^ However, the fact that only a small proportion of patients with established risk factors eventually develop HCC suggests that genetic susceptibility may play an important role in HCC development.

Our genesis constantly exposed to a variety of endogenous and exogenous factors that cause DNA damage, such as ultraviolet light, ionizing radiation and genotoxic chemicals.^9^, ^10^ Fortunately, DNA repair has lots of distinct linear pathways that can maintain genome stability while effectively preventing or repairing various types of DNA damage.^11^ Homologous recombination (HR) is a component of the DNA repair pathway, and it is essential to support DNA replication and repair DNA damage, such as DNA double–strand breaks (DSBs) and DNA cross‐links.^12^, ^13^ It has been demonstrated that various HCC–associated risk factors are able to promote DNA damages. There are several types of DNA damage and corresponding repair pathways that have been implicated in HCC such as stalled DNA replication fork by HR^16^ *RAD51D (RAD51L3)*, located in 17q12, is a member of the *RAD51* gene family which is known to be a key player in the HR pathway.^17^, ^18^ A number of studies have indicated that the genetic variations of *RAD51D* may contribute to the development of cancer, such as ovarian cancer, breast cancer, prostate cancer and colorectal cancer.^19^, ^20^ However, the association between Single–nucleotide polymorphisms (SNPs) of *RAD51D* and hereditary susceptibility of HCC has not been investigated.

SNPs are the most frequent type of genetic variation in the human genome and usually occur in noncoding regions, they can affect transcriptional regulation or posttranscriptional gene expression. MicroRNAs (miRNAs) are short noncoding single–stranded RNAs that regulate gene expression by binding to target sites in the 3′untranslated region (3′UTR) of mRNAs. Binding to the target site alters the translation efficiency and/or stability of targeted mRNAs. Many studies have linked that 3′UTR SNPs located within miRNA target sites with cancer etiology and susceptibility, which is likely the result of altered miRNA binding to target mRNAs.^27^, ^28^ Several studies suggested that several miRNAs have been implicated in DNA damage response and DNA repair. The deregulation of some of these miRNAs are involved in genomic instability and chemosensitivity of tumors.^31^, ^32^ Jen‐Wei Huang et al reported that the targeting of RAD51D by miR‐103/107 contributes to the regulation of DNA repair.^34^

In this study, we conducted a screening on *RAD51D *from the National Center for Biotechnology Information (NCBI) dbSNP database (http://www.ncbi.nlm.nih.gov/) and NIEHS SNPinfo (http://snpinfo.niehs.nih.gov/) to seek candidate SNPs in the Chinese population. Eventually, we selected two miRNA target site SNPs for this study: rs12947947 (G > A) and rs28363292 (T > G) which are SNPs located in the 3′UTR of *RAD51D*. Based on the above facts, we hypothesize that sequence variation on two selected SNPs in miRNA target sites are associated with the development of HCC. In order to confirm this hypothesis, we conducted a case‐control study examining whether these two SNPs were associated with risk of HCC development.

## 2 MATERIALS AND METHODS

### 2.1 Study population

A total of 805 cases and 846 controls were included in our hospital–based case‐control study. All participants of the cases underwent hepatic resection and were newly diagnosed as HCC patients by pathology. Cases with previous chemotherapy or radiotherapy for tumors were excluded. The controls were nontumor patients without HCC hospitalized in the same period, and frequency matched with case group by age (±5 years), gender, and nation. Cases or controls with HCV infection or other cancers were also excluded. All subjects were recruited from January 2007 to April 2011 in the First Affiliated Hospital of Guangxi Medical University, Affiliated Tumor Hospital of Guangxi Medical University and Affiliated Hospital of Guilin Medical University. All participants in the study signed informed consent. This study was approved by the Ethics Committee Review Board of Guangxi Medical University and Guilin Medical University.

### 2.2 Questionnaire survey and blood sample collection

The subjects were investigated with a uniform epidemiological questionnaire that covered the demographic characteristics (age, gender, nation), smoking, alcohol consumption, and HBV infection. Ever smokers were defined as individuals who had smoked more than 6 months continuously or cumulatively in their lifetimes; ever drinkers were defined as individuals who had drunk alcoholic beverages at least once a week for more than 6 months. HBV infection was defined as positive for HBV surface antigen (HBsAg).^35^ 5 mL of peripheral blood sample was obtained from each study object, of which 1 mL was used to detect HBV infection status. Subsequently, genomic DNA was extracted according to the phenol–chloroform method and stored at −80°C.

### 2.3 SNP selection

We used the NIEHS SNPinfo (http://snpinfo.niehs.nih.gov/) to identify the *RAD51D* functional SNPs by using these criteria: (1) SNPs were potential target sites of miRNA. (2) The minor allele frequency (MAF) > 0.05 in Chinese population. (3) The pairwise linkage disequilibrium (LD) had an r2 threshold of 0.8. As a result, we got SNPs rs12947947, rs28363277, rs28363292 (Table S1), and LD Tag SNP selection result (Figure S1). In addition, we screened the SNPs located in the 3′UTR region of *RAD51D* gene with the NCBI dbSNP database (https://www.ncbi.nlm.nih.gov/). However, We found that the rs28363277 is not in the 3′UTR region of *RAD51D*. Finally, rs12947947 (G > A) and rs28363292 (T > G) of *RAD51D* were selected for our study.

### 2.4 SNP genotyping

The Agena MassARRAY genotyping system (Agena; San Diego, CA) was used for genotyping following the manufacturer's instructions. The primers used for *RAD51D* of rs12947947 were: F 5′‐ACGTTGGATGCAGCAGCAAAGGCAAGTTAG‐3′ and R 5′‐ACGTTGGATGTGTGCATCACCATTGTGTCC‐3′. The primers used for *RAD51D* of rs28363292 were: F 5′‐ACGTTGGATGATGCTTACAGAGAGTGAGGC‐3′ and R 5′‐ACGTTGGATGACTGGTGACTACAGACGTTC‐3′. Each PCR reaction mixture (5 μL) contained 1 μL 10 ng/μL DNA template, 1.8 μL ddH2O, 0.5 μL 10x PCR Buffer (with 15 mmol/L MgCl2), 0.4 μL 25 mmol/L MgCl2, 0.1 μL 25 mmol/L dNTPs, 1 μL 0.5 μmol/L primer Mix, and 0.2 μL 5 U/μL Hot Star Taq polymerase. PCR reactions were carried out at 94°C for 15 minutes, 94°C for 45 cycles of 20 seconds, 56°C for 30 seconds, 72°C for 1 minute, and finally incubated at 72°C for 3 minutes. Two blank control wells were used in each 96‐well plate. The results of the genotyping were analyzed with the MassARRAY Typer software version 4.0.

### 2.5 Statistical analysis

The distributions of general characteristics between cases and controls were performed using chi‐square test. Hardy‐Weinberg Equilibrium (HWE) in the controls was tested using a chi‐square goodness‐of‐fit test. Logistic regression models were used to estimate adjusted odds ratio (OR) and 95% confidence interval (CI). The multivariate adjustment included age, gender, nation, smoking, drinking, and HBV infection. *P* < 0.05 was the criterion of statistical significance and all statistical tests were two‐tailed. The data were treated using SPSS 17.0 statistical software (SPSS Institute, Chicago, IL).

## 3 RESULTS

### 3.1 Characteristics of the study population

Distributions of general characteristics of the study population are presented in Table 1. In brief, there were no statistical differences in the distribution of age, gender and nation between HCC patients and control subjects (*P* > 0.05). However, the HCC patients were more likely to be smokers, drinkers and HBV infected individuals (*P* < 0.001).

**Table 1:** Distribution of general characteristics in HCC patients and control subjects

Characteristics	Cases n (%)	Controls n (%)	χ^2^	*P‐value* a
All subjects	805 (100%)	846 (100%)		
Age (years)			0.146	0.702
≦49	413 (51.30)	442 (52.25)		
>49	392 (48.70)	404 (47.75)		
Gender			2.964	0.085
Male	712 (88.45)	770 (91.02)		
Female	93 (11.55)	76 (8.98)		
Nation			5.886	0.053
Han	512 (63.60)	489 (57.80)		
Zhuang	276 (34.29)	338 (39.95)		
other	17 (2.11)	19 (2.25)		
Smoking			54.067	<0.001
No	468 (58.14)	636 (75.18)		
Yes	337 (41.86)	210 (24.82)		
Drinking			50.420	<0.001
No	506 (62.86)	666 (78.72)		
Yes	299 (37.14)	180 (21.28)		
HBV infection			937.518	<0.001
(−)	125 (15.53)	767 (90.66)		
(+)	680 (84.47)	79 (9.34)		

HBV, hepatitis B virus.

a Two–sided Chi‐square test.

### 3.2 Distribution of genotypes and risk of HCC

The genotype distributions of *RAD51D* polymorphisms and their associations with HCC risk are shown in Table 2. The genotype frequencies of rs12947947, rs28363292 in the controls obeyed HWE (χ^2^ = 2.761, *P* = 0.097; χ^2^ = 0.693, *P* = 0.405, respectively). The chi‐square test showed that the genotype distributions of the two SNPs had no significant differences in the cases and the controls (*P* > 0.05). Although none of the variant genotypes alone was associated with significantly altered risk, both the A allele of rs12947947 and the G allele of rs28363292 tended to be associated with nonsignificantly increased HCC risk (OR = 1.428, 95%CI = 0.952‐2.143 for AG/AA of rs12947947, and OR = 1.384, 95%CI = 0.903‐2.121 for GT/GG of rs28363292).

**Table 2:** Genotype frequencies of *RAD51D* polymorphisms between cases and controls and their associations with risk of HCC

Genotypes	Cases n (%)	Controls n (%)	*P* a	Adjusted OR (95% CI)^b^	*P* b
rs12947947					
GG	683 (84.84)	711 (84.04)	0.478	1.000	
AG	117 (14.53)	125 (14.78)		1.497 (0.989‐2.265)	0.056
AA	5 (0.62)	10 (1.18)		0.575 (0.099‐3.339)	0.537
AG/AA	122 (15.16)	135 (15.96)	0.653	1.428 (0.952‐2.143)	0.085
rs28363292					
TT	677 (84.10)	740 (87.47)	0.054	1.000	
GT	121 (15.03)	104 (12.29)		1.356 (0.879‐2.093)	0.171
GG	7 (0.87)	2 (0.24)		2.611 (0.241‐28.331)	0.430
GT/GG	128 (15.90)	106 (12.53)	0.050	1.384 (0.903‐2.121)	0.136
Combined risk genotypes^c^					
0 risk genotype	565 (70.19)	614 (72.58)	0.283	1.000	**0.024**
1‐2 risk genotype	240 (29.81)	232 (27.42)		**1.462 (1.050‐2.036)**	

Bold value indicates statistically significant, *P* < 0.05.

a Two‐side Chi‐square test for genotype distribution between cases and conctrols.

b Adjusted for age, gender, nation, smoking, drinking, and HBV infection in a logistic regression model.

c *RAD51D *rs12947947 AG/AA and rs28363292 GT/GG were considered as risk genotypes.

Considering the potential combined effect of *RAD51D* SNPs on risk of HCC, we combined them by the number of the putative risk genotypes (i.e., rs12947947 AG/AA and rs28363292 GT/GG) to assess their possible combined effect on HCC risk. We found that the combined genotypes with 1‐2 risk genotypes was associated with significantly increased overall risk of HCC (OR = 1.462, 95% CI = 1.050‐2.036) (Table 2).

### 3.3 Stratification analysis

In order to further identify the relationship between rs12947947, rs28363292 polymorphism and HCC risk, the dominant genetic model of the two SNPs and combined genotype were stratified by subgroups of age, gender, nation, smoking, drinking, and HBV infection. As shown in Table 3, GT/GG genotype of rs28363292 had a relationship with a significantly increased HCC risk in zhuang people (OR = 3.913, 95% CI = 1.873‐8.175) and non‐HBV infected population (OR = 1.774, 95% CI = 1.060‐2.969), compared with TT genotype. Interestingly, further analysis revealed that the combined 1‐2 risk genotypes were associated with a statistically significantly increased HCC risk in zhuang people (OR = 2.817, 95% CI = 1.532‐5.182) and non‐HBV infected population (OR = 1.567, 95%CI = 1.042‐2.358), compared with the combined genotype than with the 0 risk genotype (Table 4 **).**

**Table 3:** Stratification analyses between rs12947947, rs28363292 polymorphisms and HCC risk

Variables	rs12947947		Adjusted OR (95% CI)^a^	*P* a	rs28363292		Adjusted OR (95% CI)^a^	*P* a
	(cases/controls)				(cases/controls)			
Genotypes	GG	AG/AA			TT	GT/GG		
Age								
≤49	352/372	61/70	1.386 (0.730‐2.630)	0.318	349/382	64/60	1.146 (0.599‐2.193)	0.680
>49	331/339	61/65	1.370 (0.810‐2.315)	0.240	328/358	64/46	1.611 (0.912‐2.845)	0.100
Gender								
Female	78/66	15/10	2.667 (0.860‐8.269)	0.089	77/65	16/11	1.233 (0.367‐4.147)	0.735
Male	605/645	107/125	1.298 (0.839‐2.007)	0.241	600/675	112/95	1.425 (0.900‐2.257)	0.131
Nation								
Han	432/406	80/83	1.397 (0.856‐2.280)	0.181	431/421	81/68	0.859 (0.508‐1.454)	0.571
Zhuang	237/287	39/51	1.446 (0.692‐3.021)	0.326	229/303	47/35	**3.913 (1.873‐8.175)**	**<0.001**
Other	14/18	3/1	0.272 (0.002‐43.938)	0.616	17/16	0/3	—	—
Smoking								
No	393/537	75/99	1.660 (0.998‐2.762)	0.051	394/555	74/81	1.292 (0.751‐2.224)	0.355
Yes	290/174	47/36	1.121 (0.568‐2.210)	0.742	283/185	54/25	1.559 (0.766‐3.173)	0.221
Drinking								
No	436/565	70/101	1.410 (0.849‐2.340)	0.184	418/579	88/87	1.354 (0.812‐2.260)	0.246
Yes	247/146	52/34	1.484 (0.750‐2.935)	0.257	259/161	40/19	1.483 (0.662‐3.324)	0.339
HBV infection								
(−)	100/638	25/129	1.241 (0.758‐2.033)	0.391	101/674	24/93	**1.774 (1.060‐2.969)**	**0.029**
(+)	583/73	97/6	2.097 (0.881‐4.989)	0.094	576/66	104/13	0.940 (0.496‐1.780)	0.849

Bold value indicates statistically significant, *P* < 0.05.

a Adjusted for age, gender, nation, smoking, drinking, and HBV infection in a logistic regression model.

**Table 4:** Stratification analyses between the combined genotypes of *RAD51D* polymorphisms and HCC risk

Variables	Combined risk genotypes^a^ (cases/controls)		Adjusted OR (95% CI)^b^	*P* b
	0 risk genotype	1‐2 risk genotypes		
Age				
≤49	293/318	120/124	1.317 (0.791‐2.194)	0.290
>49	272/296	120/108	1.520 (0.984‐2.349)	0.059
Gender				
Female	63/55	30/21	2.153 (0.833‐5.563)	0.113
Male	502/559	210/211	1.387 (0.973‐1.977	0.071
Nation				
Han	357/343	155/146	1.101 (0.737‐1.645)	0.639
Zhuang	194/256	82/82	**2.817 (1.532‐5.182)**	**<0.001**
other	14/15	3/4	0.154 (0.001‐18.491)	0.444
Smoking				
No	326/463	142/173	1.502 (0.987‐2.284)	0.057
Yes	239/151	98/59	1.405 (0.813‐2.425)	0.223
Drinking				
No	354/485	152/181	1.393 (0.929‐2.088)	0.109
Yes	211/129	88/51	1.624 (0.906‐2.913)	0.104
HBV infection				
(−)	78/554	47/213	**1.567 (1.042‐2.358)**	**0.031**
(+)	487/60	193/19	1.293 (0.747‐2.240)	0.359

Bold value indicates statistically significant, *P* < 0.05.

a Risk genotypes were represented by rs12947947 AG/AA and rs28363292 GT/GG.

b Adjusted for age, gender, nation, smoking, drinking, and HBV infection in a logistic regression model.

## 4 DISCUSSION

In this study, we investigated whether *RAD51*D gene rs12947947 and rs28363292 polymorphisms are associated with the risk of HCC in the population of South China. Although we did not find two SNPs individually that had any significant main effect on risk of HCC, we did find that those who carried the 1‐2 combined risk genotypes (i.e., rs12947947 AG/AA and rs28363292 GT/GG) appeared to have an increased risk of HCC. In conclusion, we found that the two selected SNPs had a joint effect on the HCC risk.

The RAD51 protein and its paralogs (RAD51B, RAD51C, RAD51D, XRCC2 and XRCC3) are recruited to form a helical nucleofilament on the exposed single–stranded DNA (ssDNA) for the maintenance of genome stability in mammalian cells.^36^ In humans, the functions of the paralogs as mediators of HR demonstrate important tumor suppressor activity. Previous studies found some potentially functional SNPs of *RAD51 *gene family polymorphisms (eg, *RAD51*‐135 G > C, *XRCC2 *R188H, *XRCC3 *T241M) to be associated with various types of cancer risks, including prostate cancer and lung cancer, breast cancer.^37^, ^38^ RAD51D protein is believed to have ssDNA binding activity and DNA‐–timulated adenosine triphosphatase (ATPase) activity. Moreover, the complex of RAD51D and XRCC2 is likely to be important for genetic recombination and protection against DNA–damaging agents.^40^ Therefore, the mutations in *RAD51D* lead to the accumulation of unrepaired DSBs and are associated with the development of tumors. In addition, miRNAs are posttranscriptionally downregulate gene expression through translational repression and mRNA destabilization.^41^ A variety of miRNAs have been found to regulate tumorigenesis and responses to cancer treatment by inducing chemosensitization by affecting RAD51 expression in HR repair mechanisms.^42^, ^43^ miR‐103 and miR‐107 was shown to directly target and regulate RAD51D, which is critical for miR‐103/107–mediated chemosensitization.^34^ Hence, the mutations in miRNA target sites of *RAD51D* are also associated with the development of tumors. It is possible that rs12947947 and rs28363292 polymorphisms in miRNA target sites of *RAD51D* genes may jointly contribute to the risk of HCC. However, further large studies and functional studies are required to validate our findings.

The genetic variant E233G in *RAD51D* was regarded as a potential low–penetrance breast cancer allele in high‐risk, site‐specific, familial breast cancer.^44^ Aditi et al found that the *RAD51D* (E233G) breast cancer associated variant increased cell growth and cisplatin resistance dependent upon the status of the p53 gene in human breast carcinoma cell lines.^45^ Moreover, Phillip et al investigated that a p53 deletion was sufficient to extend the life span of RAD51D–deficient embryos by up to 6 days and rescued the cell lethal phenotype.^46^ The p53 tumor–suppressor gene plays a central role in regulating cell growth, DNA repair and apoptosis.^47^ Previous studies strongly demonstrated that *RAD51D* functions are monitored by p53.^48^, ^49^ However, further study of this mechanism is needed.

The occurrence of HCC is considered to be a multistage process involving multiple genetic or environmental factors. Interaction and cross‐regulation of distinct factors together promote HCC development.^5^ However, from the stratification analysis results in this study, we did not find the interaction of rs28363292 and HBV infection had any combined effect on HCC risk. Besides, we also did not find the interaction of the combined genotypes and HBV infection had any combined effect on HCC risk.

In our study, Neyman bias may be avoided by selecting newly diagnosed HCC patients as cases. However, some limitations of this study also should be considered when interpreting the results. First, our study was a hospital–based case‐control study with potential selection bias. The sample size in the stratification analyses might be relatively small, which could not provide enough statistical power. Second, more than 80% cases were HBV infected. Such results need to be confirmed in an HCV epidemic population. As a result, more diverse populations will be needed to prove the results in future studies.

## 5 CONCLUSION

To summarize, we selected two SNPs within noncoding regions of *RAD51D* and observed that none had a main effect on HCC cancer risk. However, given only a modest effect of each SNP on its own, evaluating their joint effects may help us better understand the effect of SNPs in HCC cancer. Indeed, we found that the combined genotypes of these two polymorphisms (i.e., rs12947947 AG/AA and rs28363292 GT/GG) were associated with a statistically significantly increased risk of HCC suggesting that rs12947947 and rs28363292 SNPs may jointly contribute to the risk of HCC. However, further large studies and functional studies are required to validate our findings.
