# Supplementary material for: Association between polymorphisms in MicroRNA target sites of RAD51D genes and risk of hepatocellular carcinoma
Source: Cancer Med. 2019 Mar 18;8(5):2545–52. doi: 10.1002/cam4.2068 (PMC6536933; doi:10.1002/cam4.2068)
Supplement: Supplementary file 1 [file CAM4-8-2545-s001.docx]

| Table S1 The functional SNPs of RAD51D | | | | | |  |
| --- | --- | --- | --- | --- | --- | --- |
| TagSNP | Allele | miRNA(miRanda) | miRNA(Sanger) | Allele | ave.MAF | ave.r2 |
| rs12947947 | A/G | Y | Y | G | 0.054 | 1 |
| rs28363277 | G/A | -- | Y | G | 0.119 | 1 |
| rs28363292 | T/G | Y | -- | T | 0.065 | 1 |


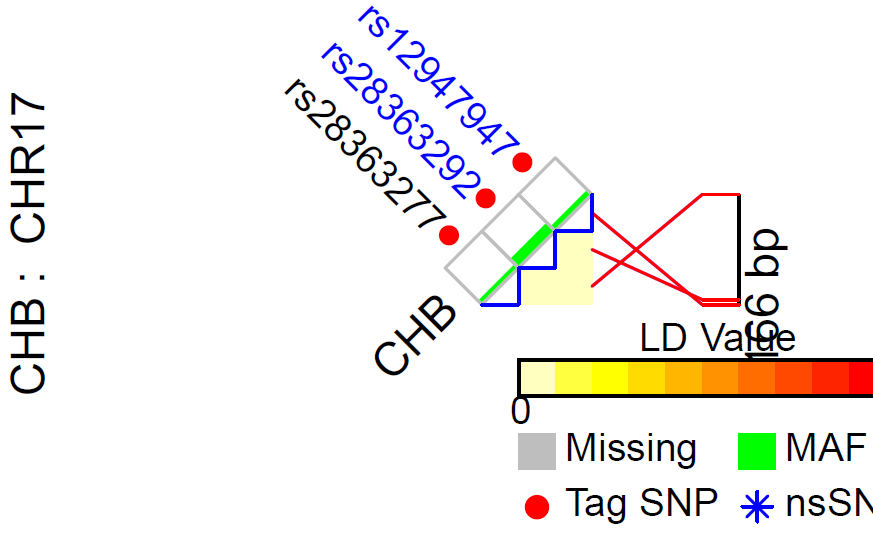


Figure S1. LD Tag SNP Selection Result
